# Nano-biomaterials and advanced fabrication techniques for engineering skeletal muscle tissue constructs in regenerative medicine

**DOI:** 10.1186/s40580-023-00398-y

**Published:** 2023-10-21

**Authors:** Seokgyu Han, Sebastián Herrera Cruz, Sungsu Park, Su Ryon Shin

**Affiliations:** 1 Division of Engineering in Medicine, Department of Medicine, Brigham and Women’s Hospital, Harvard Medical School, Cambridge, MA 02139 USA; 2 School of Mechanical Engineering, Sungkyunkwan University (SKKU), Suwon, 16419 Korea; 3 Department of Biophysics, Institute of Quantum Biophysics (IQB), Sungkyunkwan University (SKKU), Suwon, 16419 Korea

**Keywords:** Nanomaterials, Biomaterials, Stem cells, Skeletal muscle, Tissue engineering, Tissue regeneration

## Abstract

Engineered three-dimensional (3D) tissue constructs have emerged as a promising solution for regenerating damaged muscle tissue resulting from traumatic or surgical events. 3D architecture and function of the muscle tissue constructs can be customized by selecting types of biomaterials and cells that can be engineered with desired shapes and sizes through various nano- and micro-fabrication techniques. Despite significant progress in this field, further research is needed to improve, in terms of biomaterials properties and fabrication techniques, the resemblance of function and complex architecture of engineered constructs to native muscle tissues, potentially enhancing muscle tissue regeneration and restoring muscle function. In this review, we discuss the latest trends in using nano-biomaterials and advanced nano-/micro-fabrication techniques for creating 3D muscle tissue constructs and their regeneration ability. Current challenges and potential solutions are highlighted, and we discuss the implications and opportunities of a future perspective in the field, including the possibility for creating personalized and biomanufacturable platforms.

## Introduction

Volumetric muscle loss (VML) refers to a significant loss of muscle tissue due to trauma or surgery, leading to the failure of intrinsic muscle regeneration and function and the healing of the defect area by fibrosis [1]. The current gold standard for treating VML is the transplantation of functional muscle tissues obtained from donors [2]. However, tissue transplantation still possesses multiple clinical limitations, such as donor site morbidity, lack of donors, immune rejection, and low integration, which can decrease the outcomes of functional regeneration [3].

To find an alternative method, various regenerative therapies, such as stem cells delivery, scaffolds implantations, and engineered muscle tissue grafts, have been developed to address long-term functional deficits and various pathologic comorbidities caused by a large amount of tissue loss [4, 5]. These therapies aim to regenerate new muscle via implanted stem cells or by inducing the differentiation of host cells along with neuromuscular junctions and blood vessels, which are necessary to restore the muscle function [6, 7]. Currently, these approaches have been combined with novel engineering strategies, such as bioprinting, which enables the precise control over the size, shape, and compartmentation of various cells, ultimately providing personalized engineered tissues with complex 3D architectures to fit defect areas [8]. Furthermore, many studies have explored nanomaterials and nanotechnologies to resemble the biophysical and biological properties of the nanofibrous native extracellular matrix (ECM) that allow us to create functional tissue constructs, like the native skeletal muscle tissue [9]. Incorporation of these unique nanomaterials or nanostructures into 3D engineered tissue constructs using advanced bioprinting technologies can improve the communication between cells and promote the integration of the engineered tissue with the host tissue, ultimately leading to better outcomes for regeneration.

**Fig. 1:**
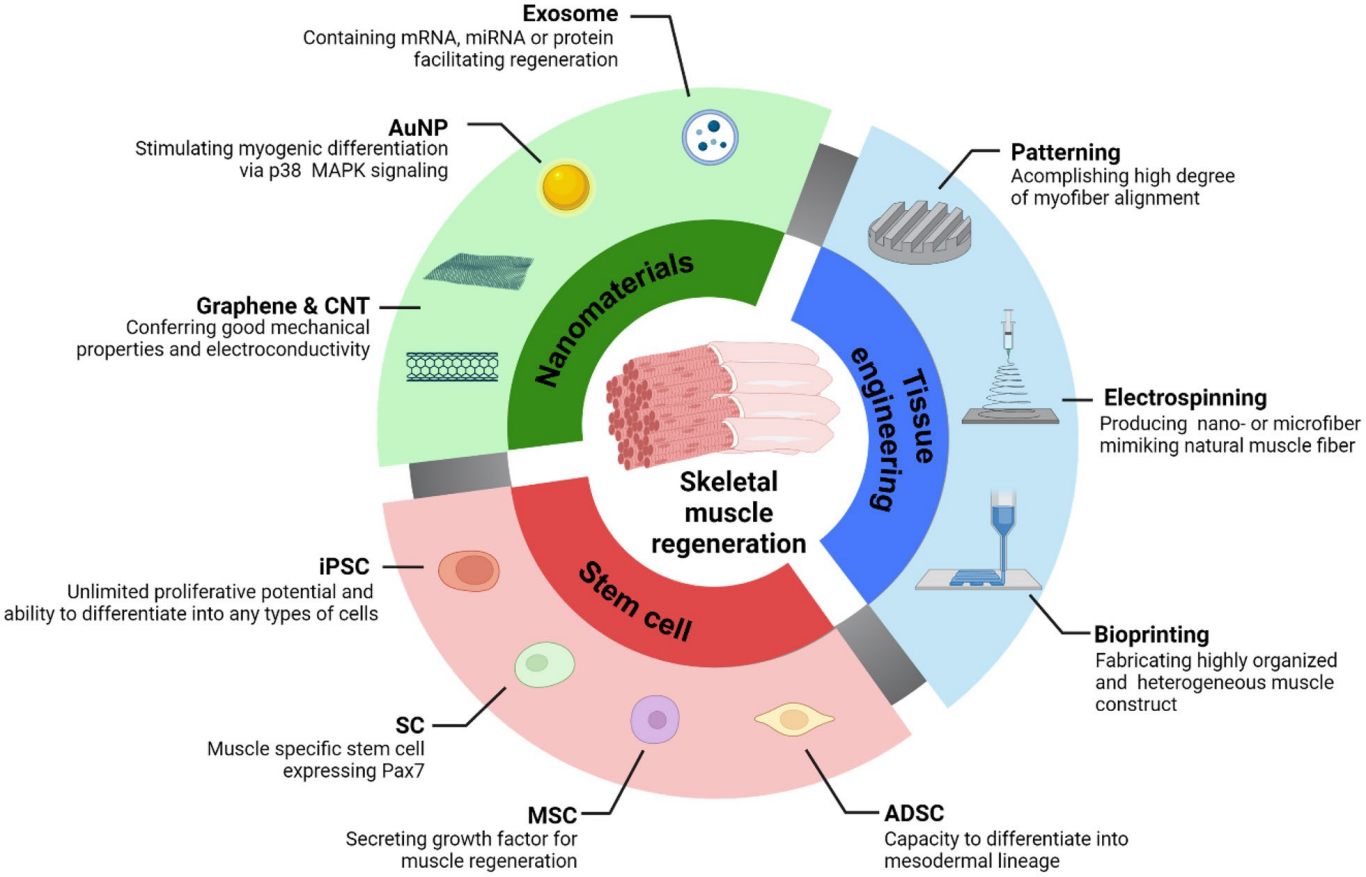
Stem cells, nanomaterials, and advanced tissue engineering for engineering skeletal muscle tissue constructs in regenerative medicine. (*AuNP* Gold nanoparticle, *CNT* Carbon nanotube, *iPSC* Induced pluripotent stem cell, *ADSC* Adipose-derived stem cell, *MSC* Mesenchymal stem cell)

Previous reviews have primarily focused on muscle regeneration using stem cells and biomaterials as scaffolds [4, 10–13]. This review aims to address the existing limitations in the literature by providing an overview of recent advancements in the combined utilization of stem cells, nano-biomaterials, and nano/micro fabrication technologies in bioengineering strategies for muscle regeneration. It also explores the diverse roles played by various nanomaterials in this field (Fig. 1). Finally, we will provide future perspectives on skeletal muscle regeneration.

## Stem cells for skeletal muscle regeneration

Most of the cells in skeletal muscle are multinucleated, generating myofibers that are surrounded by ECM such as endomysium. ECM components, such as collagen (types I, III, and VI), proteoglycans, and fibronectins, exist between myofibers, regulating muscle development and facilitating the transmission of mechanical forces [14]. When the skeletal muscle undergoes some defects, the process of muscle regeneration starts with clearance of necrotic cells by phagocytosis of pro-inflammatory macrophages. The prompt clearance of debris is crucial for the timely initiation of muscle regeneration [15, 16]. Then, myofiber regeneration may be facilitated when implanted stem cells become active due to their regenerative capacity [17]. Stem cells that are introduced into the injured area can release various factors, such as cytokines, extracellular vesicles, including exosomes, and growth factors, which can affect the behavior of host cells, including resident stem cells, immune cells, and endothelial cells, etc. Specifically, these secreted factors can promote the activation of anti-inflammatory macrophages, which can help reduce inflammation and support the regeneration of muscle tissue [18]. Additionally, stem cells can also inhibit the activation of pro-inflammatory cells, further reducing inflammation and promoting tissue healing. These immunomodulatory effects play a crucial role in promoting myofiber regeneration and restoring muscle function [19]. Table 1 presents a summary of the advantages and limitations associated with these stem cell types.

**Table 1:** Characteristics of stem cells delivered for skeletal muscle regeneration

Stem cell	Characteristics	Animal model	Drawbacks	Refs.
SCs	- Muscle-specific stem cells - Expression of Pax7 - Ability to differentiate into multiple types of muscle cells	Mice cardiotoxin tibialis anterior (TA)	- Limited numbers of cells - Difficulty to obtain from samples - Loss of regenerative ability over time	[24, 25, 154]
ADSCs	- Paracrine effect for muscle regeneration - Capacity to differentiate into mesodermal lineage - Promotion of angiogenesis	Rat VML TA	- No direct evidence of myofibers differentiation after implantation	[26, 28–32]
MSCs	- Secretion of growth factor for promotion of the regeneration - Increase of myogenin expression	Mice ischemia hindlimb	- Not always differentiate into the specific type of muscle cell - Possibility to cause immune reactions - Increase of risks of cancers	[37–40]
hiPSCs	- Unlimited proliferative potential - Ability to differentiate into any types of cells - Patient-specific	Mice cardiotoxin TA	- Limited migrations from the site of injections	[11, 41–45]

Skeletal muscles have a strong regenerative ability compared to other adult tissue, partly due to the presence of satellite cells, which constitute less than 5% of the cells in muscle tissue [20]. These satellite cells rely on the transcription factor Pax7 for their proper functioning and maintenance, essential for self-regeneration [21]. Studies have shown that when Pax7 is absent, satellite cells and myoblasts experience cell cycle arrest and an imbalance in myogenic regulatory factors, underscoring the critical role of Pax7 in skeletal muscle regeneration [22]. Due to their high rate of proliferation and capacity to differentiate into a variety of muscle cell types, satellite cells have been intensively researched for muscle regeneration [23–25]. However, there are still several challenges to address in order to fully harness the potential of satellite cells in skeletal muscle regeneration. The limited number of satellite cells within muscle tissue may restrict their ability to completely repair significant or extensive muscle injuries. Obtaining an adequate quantity of satellite cells from a small muscle sample can also present difficulties, and obtaining a larger sample is not feasible due to potential harm to the biopsy site. Moreover, as satellite cells age, their ability to divide and contribute to muscle growth diminishes over time. Therefore, further research is necessary to comprehend the mechanisms underlying immune rejection and to develop strategies to mitigate the immune response to transplanted cells, which remains a significant obstacle.

Future directions for stem cell research in skeletal muscle regeneration include exploring novel delivery strategies for satellite cells, such as gene therapy and tissue engineering approaches, to enhance their survival and integration into host tissue. Researchers may also investigate the use of combination therapies that include stem cells and other growth factors or biomaterials to further enhance muscle regeneration. Finally, the development of new technologies, such as organ-on-a-chip platforms, may provide new avenues for testing the efficacy of satellite cell-based therapies in a more physiologically relevant setting.

Adipose-derived stem cells (ADSCs) are a prevalent, multipotent, adult mesenchymal stem cell type that may develop into tissues of the mesodermal lineage, such as cartilage, bone, adipose tissue, and skeletal muscle. Transplantation of ADSCs has been shown to lead to improved muscle strength and endurance in dystrophin-deficient mice [26, 27], as well as to promote the rapid onset of angiogenesis. Interestingly, ADSCs' exosomes have also been found to promote the proliferation and expression of myogenic genes [28, 29]. Previous studies have suggested that ADSCs may have the ability to differentiate into muscle cells, but in the specific study being discussed, no direct evidence of implanted ADSCs becoming new muscle fibers was observed [30]. This suggests that ADSCs may instead exert their effects through a paracrine mechanism, releasing molecules that support muscle growth and repair, rather than becoming muscle cells themselves [31, 32].

Although mesenchymal stem cells (MSCs) have demonstrated potential in promoting skeletal muscle regeneration by secreting growth factors and differentiating into skeletal muscle cells in various studies [33–38], one notable drawback is that they may not consistently differentiate into the specific type of muscle cell needed for regeneration [39]. Furthermore, there are concerns regarding potential immune reactions and an elevated risk of cancer [40]. Further research is needed to fully understand the capabilities and limitations of MSCs in muscle regeneration.

Human pluripotent stem cells (hPSCs), including human embryonic stem cells (hESCs) and induced pluripotent stem cells (hiPSCs), possess two key characteristics that differentiate them from adult stem cells. Firstly, they have an unlimited capacity to divide and create more cells, referred to as "unlimited proliferative potential." Secondly, they have the ability to differentiate into any type of cell in the body, including skeletal muscle cells (SMCs) [11]. These cells can then be expanded in the laboratory settings, and, upon transplantation into native muscle, can populate the stem cell niche and contribute to muscle repair and regeneration [41]. Numerous studies have demonstrated that transplanting hPSC-derived myogenic cells may cause them to merge with host muscle fibers and thereby improve muscular function [41–45]. The mere engraftment of myofibers alone is insufficient for muscle restoration, highlighting the need for immune modulation and the release of biological factors from implanted stem cells to enhance the regenerative process [46, 47]. The use of iPSCs to create patient-specific in vitro skeletal models is another advantage. This allows for the study of the pathogenesis of muscle diseases and the screening of potential drugs in a personalized manner. IPSCs are a powerful tool for understanding the underlying mechanisms of disease and developing therapies tailored to specific patient populations [48].

## Advantages of acellular and cellular biomaterials and their limitations

Biomaterials play a crucial role in skeletal muscle regeneration by providing 3D scaffolds for muscle tissue growth at the defect area. The scaffold provides favorable microenvironments that physically support cell attachment, proliferation, and differentiation, serving as a substrate for muscle tissue growth. Biomaterials can be engineered to deliver diverse biological factors, such as cells, growth factors, drugs, miRNAs, and other molecules, directly to the site of injury. This targeted delivery enhances the healing process and facilitates the formation of new muscle tissue, promoting muscle regeneration. Moreover, biomaterials can be designed to mimic the structure and mechanical properties of native muscle tissue, guiding the growth and organization of new muscle fibers and the maturation of newly formed muscle fibers. In cases of large-sized muscle defects, biomaterials can fill the defects and prevent the formation of fibrotic tissue that would natively occur, filling the voids of the defect spaces. This approach can improve the overall appearance of the muscle and prevent functional limitations resulting from the loss of muscle tissue. Therefore, biomaterials hold great promise in the field of skeletal muscle regeneration, as they can serve as a critical tool in repairing and restoring damaged muscle tissue. In this section, we will introduce the types of acellular and cellular biomaterials as scaffolds, delineated in Table 2, and discuss their regenerative characteristics when implanted into animal models.

**Table 2:** Characteristics of biomaterials and their combination with stem cell

	Biomaterial	Cell	Animal model	Growth factors	Structure	Characteristics	Refs.
Acellular	Alginate	–	Mouse ischemia hindlimb	VEGF, IGF-2	Bulk hydrogel	Reaching normal tissue perfusion levels in 3 weeks Regenerating damaged axons in rats with ischemic injury	[49]
			Mouse ischemia hindlimb	–	Bulk hydrogel	Minimizing s invasive surgeries shape-memory alginate	[77]
	Collagen		Rabbit acute soft tissue trauma TA	VEGF	Bulk hydrogel	Increasing muscle strength	[50]
	Skeletal muscle-ECM		Rat VML latissimus dorsi	–	Bulk dECM	Compatible with host tissue Induced myogenesis increasing mechanical stability between damaged areas	[66]
	Musculofascial-ECM		Rat VML quadricep	–	Bulk dECM	20 times higher than muscle ECM in Young’s modulus improving in myogenic properties	[69]
Cellular	Alginate	Myoblast	Mouse myotoxin/ ischemia hindlimb	VEGF, IGF-1	Bulk hydrogel	Increasing muscle regeneration by sustained release of GF	[75]
	Skeletal muscle-ECM	MSCs	Rat VML lateral gastrocnemius	–	Bulk dECM	Regenerating blood vessels and skeletal myofibers than the ECM without cells	[79]

Various biomaterials such as alginate, gelatin, and collagens have been used to engineer 3D acellular scaffolds [49, 50]. These materials offer unique properties that make them suitable for tissue engineering applications. In terms of collagens, they are vital components in many biological structures. Collagen type I stands out as the most abundant component in muscle connective tissue. Specifically, collagen type I α1 provides tensile strength and rigidity to tissue, while collagen type VI plays a crucial role in regulating satellite cell self-renewal [14, 51]. Although gelatin is a desaturated collagen; it does not provide similar biological properties to collagen and to collagen’s fibrous triplex helix structure. However, depending on the hydrolysis method, gelatin still possesses peptides and proteins that might be broken down during hydrolysis, such as cell binding sites [52]. Also, growth factors such as vascular endothelial cell growth factor (VEGF) and insulin-like growth factor-2 (IGF-2) have been combined with biomaterials that improve blood vessel perfusion, regenerate damaged axons and increase muscle strength. For instance, when an acellular collagen sponge was inserted into the vastus lateralis of a rabbit leg with a muscle defect, the number, thickness, and length of myofibers increased compared to the untreated area, and the concavity decreased by filling of the void muscle area by the scaffold. However, conventional biomaterials are often insufficient to mimic tissue microenvironment due to lack of growth factors and cytokines for cell proliferation and regeneration [53].

Decellularized extracellular matrix (dECM) scaffolds provide an attractive way to overcome the hurdles of natural and synthetic biomaterials-based scaffolds. Compared to transplanted cell-laden tissue, dECM scaffolds have superior biological properties, with a lower risk of immune response due to the removal of almost all cellular DNA [54]. Therefore, dECM scaffolds provide a promising strategy for creating a natural cell environment and maintaining various bioactive components that more closely resembles native ECM [55, 56]. Furthermore, dECM scaffolds have successfully regenerated damaged muscle tissue as they contain crucial growth factors and cytokines, such as transforming growth factor-beta (TGF-β), VEGF, fibroblast growth factor (FGF) and IGF-1 [57]. However, dECM scaffolds have several limitations, such as uncontrolled degradation and inadequate mechanical properties. Also, it is challenging to select the type of cell, tissue, and donor to obtain dECM scaffolds for treating specific types of tissues defects or diseases. ECM components of dECM scaffolds are significantly affected by the source of material such as type of cells, tissue species of donor tissue, etc. For instance, fibroblast-derived dECM is primarily composed of fibronectin and collagen I, while adipose cell-derived dECM contains collagen enriched with VEGF [58, 59]. Lung dECM is characterized by the presence of collagen, glycosaminoglycans, and elastin [60]. In contrast, kidney dECM contains glycosaminoglycans along with VEGF and bFGF [61]. Selecting the right tissue or organ for decellularization is challenging because of the need to keep its natural properties, remove all cells without causing immune reactions, and ensure its structure remains intact. Sterilization methods must also be chosen carefully to maintain the tissue's safety and function. Each tissue has its own unique requirements and challenges for regenerative applications [62]. Sometimes, dense dECM can hinder cell infiltration and impair tissue regeneration. Also, it is difficult to create complex 3D architectures to resemble the structure of native ECM due to inadequate rheological properties of dECM biomaterials using biofabrication methods. So, to fabricate large-scale and free-standing 3D scaffolds by microfabrication techniques (i.e., bioprinting), naturally derived or synthetic biomaterials have been applied for tuning the physical and rheological properties of dECM biomaterials [63]. Finally, dECM-derived biomaterials obtained from various types of tissues, such as small intestinal submucosa, dermis, or skeletal muscle tissue, through a decellularization process, can be made into 3D scaffolds using various microfabrication techniques [64, 65]. Muscle dECM-based scaffolds are compatible with host tissue and play a role as physical bridges that help with force transmission and improve muscle function by increasing mechanical stability between damaged areas [66]. The muscle-dECM, which contains components like proteoglycans and laminin, has been shown to promote the differentiation of satellite cells and their fusion into mature myofiber [67, 68]. Additionally, there is a growing interest in fascial ECM scaffolds for skeletal muscle regeneration. Fascial tissue, as a connective tissue responsible for force transmission and physical support, can serve as a template for guiding muscle tissue regeneration. Fascial dECM scaffolds offer a promising approach in this regard [69]. Furthermore, dECM-derived biomaterials can be fabricated into hydrogels. Upon implantation at the defective site, these hydrogels facilitate the infiltration of host cells and promote increased myogenesis, or the formation of new muscle tissue [70, 71]. Despite recent advancements in acellular scaffolds that fill muscle defects and provide favorable microenvironments for neo-tissue formation, complete regeneration of volumetric muscle tissue and restoration of muscle function remain challenging. However, the incorporation of myogenic precursor cells or stem cells into biomaterials holds promise in overcoming these challenges, as they possess remarkable regenerative abilities.

Cell-laden biomaterials have been shown to restore both morphology and function in the area where VML occurred [72, 73]. Moreover, combining growth factors with the scaffold has been attempted to increase the regeneration of cells in the scaffold and host tissue [74, 75]. Research has also been conducted on muscle regeneration based on the types of stem cells and scaffolds, as well as applied mechanical stimulation [76–78]. Even when a scaffold containing non-muscle-derived stem cells was implanted, the effects of muscle regeneration were observed [79]. For instance, when bone marrow derived MSCs with skeletal muscle dECM hydrogels were implanted into the lateral gastrocnemius in a VML rat model, increased von Willebrand factor stained blood vessels and recovery in the tension forces of LGAS resulted, as compared to observations in the muscles implanted with dECM without cells. Pre-implantation of cells isolated from the tibialis anterior (TA) of rats in a bladder acellular matrix with uniaxial tension using a bioreactor has also induced enhanced functional recovery in a mouse latissimus dorsi (LD) VML model [80, 81]. Continued exploration of cellular biomaterials and their applications in skeletal muscle regeneration will undoubtedly yield exciting developments and insights in the coming years. One of the key limitations and biosafety concerns associated with the use of stem cells in the clinical field is the potential for tumorigenicity [82]. Stem cells are highly beneficial for tissue engineering and regenerative medicine because they can self-renew and differentiate into various kinds of cells. However, this same property can lead to the formation of tumors if the transplanted stem cells undergo uncontrolled growth. To prevent the tumorigenesis in stem cells transplantation, stem cells should be genetically screened to define cell fate and change the cancer-related genes through epigenic modifications [83].

## Nanotechnology for muscle regeneration

In recent research on developing advanced biomaterials, the application of nanotechnology, including providing precise control over surface characteristics, has become essential because it allows for creating scaffolds that closely resemble the structures and mechanical properties of the native ECM [84]. This engineering of ECM-mimicking structures promotes cellular adhesion, proliferation, and differentiation, enhancing muscle tissue regeneration. Preferentially, the term “nanomaterials” or “nano-sized materials” refers to substances with at least one dimension less than 100 nm [85]. Nanomaterials can be utilized as carriers for the targeted and controlled release of growth factors, cytokines, and other bioactive molecules. This localized delivery enhances muscle regeneration by promoting angiogenesis, reducing inflammation, and stimulating myogenesis. Furthermore, the unique physical, mechanical, and electrical properties of nanomaterials can overcome limitations of conventional biomaterials, such as lack of mechanical strength, electrical conductivity, and nanofibrous morphology. These enhanced properties of hybrid materials improve cellular behaviors and control the differentiation of stem cells and their maturation. Moreover, nanotopography achieved through nanostructured surfaces can influence cell behavior, effectively guiding the alignment and maturation of muscle cells [86]. Nanopatterned substrates facilitate myoblast alignment, myotube formation, and the development of functional muscle tissue constructs. Additionally, hybrid materials that combine different nanoscale components show promise in terms of mechanical reinforcement, controlled release capabilities, and improved cellular interactions for muscle tissue engineering [87]. The following sections provide recent advances in nanotechnology for each respective application area (Table 3).

**Table 3:** Nanomaterials for muscle regeneration

	Materials	Cell	Animal model	3D structure	Feature	Refs.
CNT	Polydopamine coated CNT/Gelatin	C2C12	Rat VML TA	Tubular cryogel	Inducing myogenic differentiation of C2C12 and muscle regeneration by polydopamine coated CNT	[90]
Graphene	Polydopamine/rGO aerogel	C2C12	Mouse denervated gastrocnemius muscle	Bulk aerogel	Promoting the weight, fiber size, and contractile force of the denervated muscles	[95]
	Polycitrate-based rGO	C2C12	Rat VML TA	Rectangle film	Fabricating highly conductive and elastomeric and enhancing myogenic gene expression	[97]
Exosome	Myostatin propeptide conjugated exosome	–	mdx mouse cardiotoxin TA	–	Increasing muscle mass and functional rescue without any detectable toxicity in mdx mice	[102]
PLGA-PEG	PTEN inhibitor	C2C12	–	–	Promoting the selective uptake by muscle cells/tissue in vitro and in vivo	[104]
AuNP	IL-4 or IL-10	–	mdx mouse VML TA	–	Improving muscle function in murine DMD model	[105]
	F-127-polydopamine NP	C2C12	Rat VML TA	Bulk hydrogel	Regenerating structural and functional in the VML mouse model	[107]

**Fig. 2:**
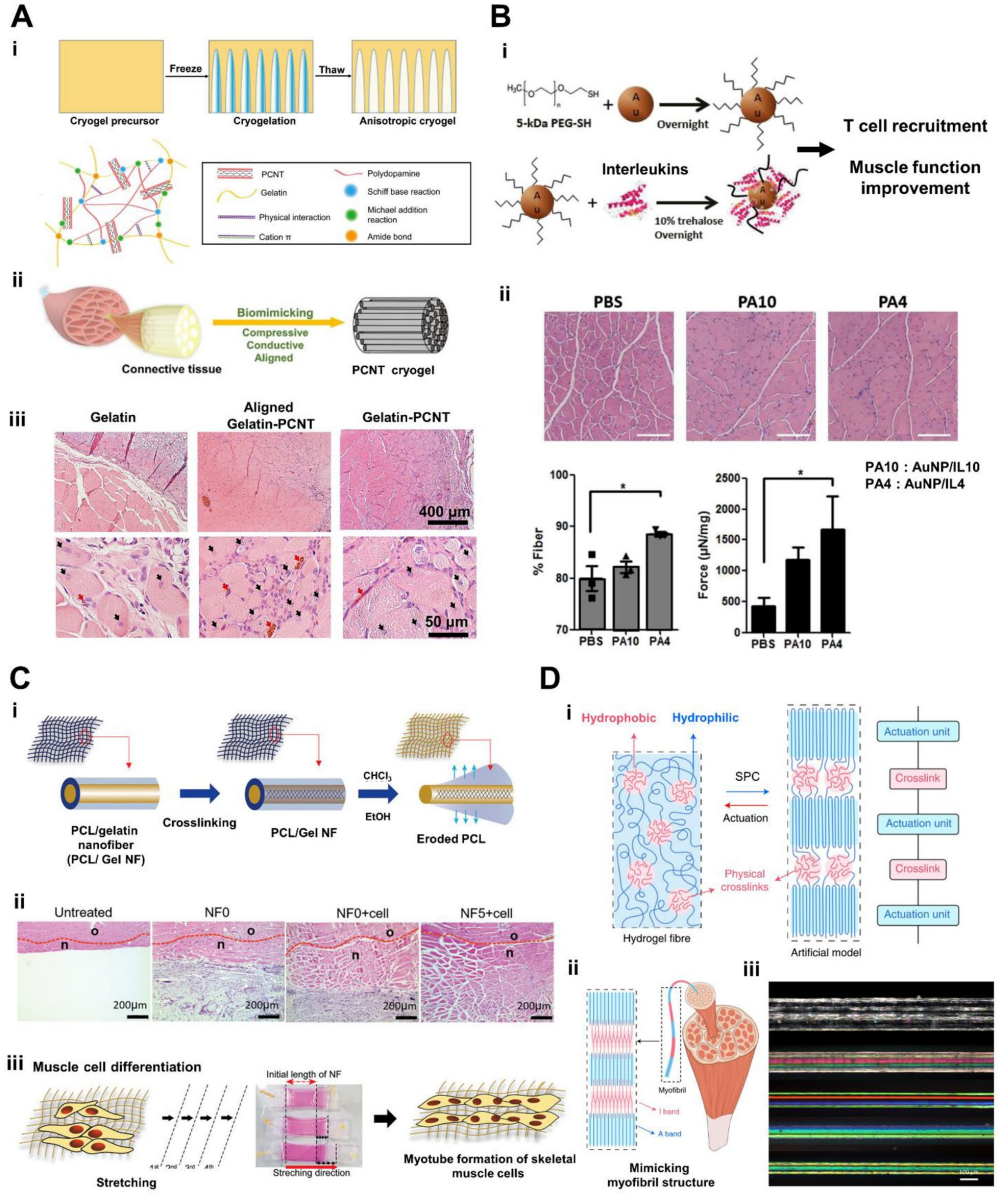
Nanomaterials and nanostructures for engineering skeletal muscle tissues and improving muscle regeneration. **A**. 3D Anisotropic cryogels composed of conductive aligned polydopamine coated carbon nanotubes (PCNTs) for muscle regeneration. (i) Schematic of the fabrication of PCNT cryogel [90]. (ii) Compressive, conductive and highly aligned skeletal muscle PCNT cryogel mimicking mechanical properties of natural muscle and inducing cell alignment and differentiation. (iii) Evaluation of in vivo muscle regeneration in a rat TA muscle defect model after implantation of the PCNT cryogel for 4 weeks. Red arrows indicate the presence of freshly created blood vessels, while black arrows indicate the newly formed muscle fibers assessed by centronucleated myofibers. **B**. Anti-inflammatory cytokine immobilized AuNPs for improving muscle function in dystrophic mice. (i) Schematic showing PEGylation and interleukin-4 (IL-4) conjugations to AuNPs for T cell recruitment and muscle function improvement. (ii) Enhancement of muscle functions observed in mdx mice by IL 4-conjugated AuNPs. Scale bar: 300 μm [105]. **C**. Stretchable nanofibrous sheet using coaxial electrospinning for improving muscle regeneration. (i) Schematic showing co-axial electrospinning of PCL and gelatin solutions, followed by chemical crosslinking of the gelatin core using glutaraldehyde. The sacrificial PCL layers were removed to produce gelatin co-axial nanofibers (NF). (ii) NF5 + C2C12 (5% stretched nanofiber with cell) showed the largest muscle regeneration compared to NF0 + cell (unstretched nanofiber with cell) or NF0 (unstretched nanofiber) (n: interface between host tissue and implants. o: host muscle tissue) [111]. (iii) Stretchable nanofiber for enhancing myotube formation. **D**. Nanostructured fibers resembling the ordered and striated pattern of myofibrils via self-assembly of ABA triblock copolymers.. (i) Schematic showing the fiber fabrication process and structural characteristics of the fiber. (ii) Nanostructured fiber mimicking the patterns (A and I band) and the size of myofibril. (iii) Images showing elongation ratios ranging from one to five. As the elongation ratio increased, the diameters of the subsequently treated fibers rapidly decreased [112]

Carbon nanotubes (CNTs) are cylindrical carbon tubes with high aspect ratios and nanometer-diameters that can be significantly longer than 100 nm [88]. They possess superior mechanical properties, large surface areas, and electrical conductivity properties. In a study by Ramón-Azcón et al. [89], dielectrophoresis was used to align multi-walled CNTs within GelMA hydrogel. It was observed that the electrical conductivity of the GelMA hydrogel increased as a result, leading to elevated gene expression of myogenic differentiation markers (such as sarcomeric actin and myogenin) in C2C12 myoblasts. This effect was not observed with randomly dispersed CNTs within GelMA hydrogel, indicating the enhanced efficiency of electrical stimulation due to alignment. Incorporating polydopamine-coated CNTs into gelatin-based cryogels promoted muscle regeneration in a rat TA muscle defect model. This enhancement was attributed to the improved mechanical properties and conductivity of the cryogels, which facilitated enhanced muscle cell communication and signal transduction (Fig. 2A) [90].

Graphene is an allotrope of carbon that exists in a 2D monolayer with a honeycomb pattern and has π-π bonds between carbon layers. The distance between carbon atoms in their hexagonal framework is around 140 nm, and the integral strong covalent bonding allows the structure of graphene to maintain a few hundred-folds higher tensile strength than steel [91]. Graphene oxide (GO) is an oxidized form of graphene having functional groups that include oxygen, such as hydroxyl, carboxyl, and epoxy carbonyl, making the final substance water-dispersible [92]. C2C12 myoblasts cultured on GO substrate significantly increased their myogenic proteins, such as myosin heavy chain and myogenin [93]. The heightened myogenic behavior observed on GO surfaces can be attributed to the surface oxygen concentration and roughness, which have an impact on serum protein adsorption. Moreover, highly wrinkled GO substrates were found to promote greater cell adhesion regions and more efficient myogenic differentiation [94]. Wang et al. [95] fabricated ultralight, conductive, and elastic aerogels using polydopamine and reduced GO (rGO), which enabled the promotion of fiber size and contractile forces of the denervated muscle. Annabi et al*.* [96] developed a conductive hydrogel by combining GO with the elastic natural material tropoelastin, which resulted in a hydrogel with 250% ultimate strain and 9700° of reversible rotation. The added electric conductivity of GO allowed for muscle contraction with low voltage after implantation of the hydrogel in rat abdominal muscle. Similarly, Du et al*.* [97] added rGO to poly(citric acid-octanediol-polyethylene glycol) (PCE) to provide conductivity, and they observed improved expression levels of MyoD, myogenin, and Troponin T, as well as the development of new muscle tissue and an increase in the mass of centronucleated myofibers and capillaries within a week.

Other nanoparticles, such as exosomes and gold nanoparticles (AuNPs), have been used in skeletal muscle regeneration. They can be used in combination with proteins for targeted delivery as they have unique properties. For instance, AuNPs exhibit high surface activity, strong antioxidant properties, and good biocompatibility, while exosomes possess innate stability, low immunogenicity, and excellent cell penetration capacity [98, 99]. Exosomes, a natural biological nanoparticle, contain messenger RNA, microRNA, or other proteins, and act as messengers to transfer these proteins to other cells [100]. MSC-derived exosomes have been found to promote myogenesis in both in vitro and in vivo studies, potentially mediated by the presence of miRNA molecules. For instance, miR-494, found in MSC-exosomes, has been shown to enhance regeneration processes [101]. Ran et al*.* [102] anchored myostatin propeptide, a negative regulator of muscle growth [103], into the CD63 loop. This inhibited myostatin activity and was observed to have beneficial effects in mdx mice [102]. Huang et al*.* [104] used M12-conjugated poly(lactic-co-glycolic acid)-polyethylene glycol (PLGA-PEG) to selectively deliver phosphatase and tensin homolog inhibitors to muscle cells in vivo, leading to improved muscle growth. AuNPs were employed to conjugate anti-inflammatory cytokines, such as IL-4, for the purpose of enhancing muscle function in mdx mouse models, through immune cell recruitment [105] (Fig. 2B). Ge et al*.* [106, 107] demonstrated that AuNPs can stimulate myogenic differentiation via p38 mitogen-activated protein kinase (MAPK) signaling. Furthermore, they found that combining AuNPs with hydrogels and injecting them into rat muscle defect models facilitated muscle tissue formation.

Nanomaterials, including GO, have been employed in tissue engineering to fabricate skeletal muscle constructs. A summary of these applications can be found in Table 4. Kim et al*.* [108] used graphene to create stretchable and implantable electric devices using patterning. Graphene was capable of regulating the proliferation and differentiation of C2C12 myoblasts and reading electromyographical signals when implanted. Patel et al*.* [109] used CNTs to create nano-functionalized foam scaffolds via a pyrolysis technique, and the CNTs were then aligned to significantly increase the fusion of C2C12 myoblasts into multinucleated myotubes. Park et al*.* [110] used femtosecond laser ablation to create line patterns on GO-incorporated polyacrylamide hydrogel. When C2C12 myoblasts were cultured on these macro-patterned substrates and subjected to electrical stimulation, they exhibited improved myogenesis and increased differentiation. Electrospinning, a technique capable of producing nanofibers ranging from 100 nm to several micrometers in diameter, was utilized in combination with nanomaterials like GO to fabricate muscle constructs using C2C12 myoblasts [111] (Fig. 2C). Lang et al*.* [112] developed myofibril-resembling fibers with I and A band patterns using poly(styrene)-b-poly(ethylene oxide)-b-poly(styrene) through the solvent injection technique. These fibers demonstrated superior efficiency, actuation strain, and mechanical properties compared to existing actuators (Fig. 2D).

**Table 4:** Technique for fabricating nanostructure

Technique	Materials	Cell	Animal model	3D structure	Feature (Add size in the feature)	Refs.
Electrospinning	PCL/gelatin	C2C12	Mouse VML quadriceps	Fiber	Enhancing myotube formation on the nanofiber scaffold	[111]
Patterning	Graphene		Mouse VML hind limb	Mesh pattern	Stimulating implanted sites electrically and/or optically in vivo and recoding electromyographical signals	[108]
Pyrolysis	Nanostructured CNT		–	Foam, fiber	Enhancing myocyte fusion into multinucleated mature myotubes	[109]
Laser ablation	GO/polyacrylamide		–	Line pattern	Micropatterned rGO/PAAm hydrogel enhancing myogenesis and increased differentiation	[110]
Solvent injection	poly(styrene)-b-poly(ethylene oxide)-b-poly(styrene)	–	–	Fiber	Resembling myofibril (I, A band) and excelling in efficiency, actuation strain and mechanical properties over current actuators	[112]

Overall, nanomaterials hold great potential as a tool for enhancing skeletal muscle regeneration due to their unique mechanical and electrical properties or delivery of biological factors. However, it is worth noting that most in vitro experiments have used C2C12 myoblasts, an immortalized cell line, rather than primary cells or stem cells [12]. Further research utilizing primary cells and stem cells is essential to gain a comprehensive understanding of the effectiveness and safety of these materials, paving the way for their translation into clinical practice. Additionally, it is crucial to address the long-term safety issues and limitations associated with non-biodegradable nanomaterials, such as bioaccumulation, long-term exposure effects, and off-target effects [113].

## Engineering 3D muscle tissues for skeletal muscle regeneration

The fabrication of functional 3D muscle tissues by tissue engineering offers significant potential as an alternative therapy since it may restore the structure and function of damaged muscle tissue. Accelerated muscle tissue formation and integration can be achieved by conferring constructs with biomimetic physical properties and architectures and integrating skeletal muscle and other cells, such as endothelial cells and neurons. To mimic highly aligned muscle fibers and organized vessel networks like native muscle tissue, various nano- and micro-fabrication techniques have been developed. Bioprinting, including in-situ and ex-situ, and electrospinning technologies have been used to integrate and implant different types of cells in a scalable manner. Furthermore, hydrogel-based bioinks can be fortified with specific nanomaterials, such as GO, AuNPs, laponite and CNTs to enhance their printability and mechanical properties [114]. These diverse nanomaterials not only provide mechanical reinforcement but also introduce unique functionalities, like pH-responsiveness and electro-conductivity. Furthermore, nanomaterials enable targeted and controlled delivery of various biomolecules, including miRNA, proteins or drugs, resulted in improving biological properties of the bioinks. These hybrid bioinks closely mimic the anatomy and function of native extracellular matrix to improve tissue-engineered muscle performance and regeneration. Table 5 summarizes the investigation and findings on the utilization of various printing techniques, along with nano-biomaterials and stem cells, for muscle regeneration in the context of tissue engineering.

**Table 5:** Bioprinting for generation of functional skeletal muscle construct

Printing methods	Materials	Cells	Animal model	Features	Refs.
Inkjet printing	Fib/BMP-2	hMPCs	–	Controlling cells fate of primary muscle derived stem cells toward osteogenic or myogenic cells by BMP-2 patterning	[118]
3D ITOP	Fib/gelatin/ HA/glycerol	hMPCs	Rat VML TA	Fabricating biomimetic implantable human skeletal muscle construct (mm^3^-cm^3^ size) made up of hundreds of long parallel myofiber bundles	[120]
	Fib/gelatin/ HA/glycerol	hMPCs/hNSCs	Rat VML TA	Forming neuro-muscular junction and facilitating rapid innervations in rodent model	[121]
	Fib/NF loaded microsphere	hMPCs	–	Accelerating peripheral nerve regeneration and innervation by release NF delivery	[122]
Extrusion	Au nanowires/collagen	C2C12	Rat VML temporalis muscle	Increasing C2C12 differentiation by aligned in Au nanowires	[130]
	Collagen	hADSC/HUVEC	Rat VML temporalis muscle	Achieving *in-situ* direct bioprinting by combining bioprinting with bioreactor enabling	[145]
	dECM	–	Canine VML biceps femoris	Acellular 3D bioprinted dECM patches for implantation of bulk patient-specific scaffold (12 × 8 × 2 cm) based on computed tomography imaging	[127]
	Collagen	hMSCs	Rat VML TA	Fabricating collagen microfiber with 1,000 times higher tensile strengths than a normal collagen gel	[124]
SLA	Oxidized methacrylic alginate/PEGMA	Primary neurons/C2C12	–	Demonstrating spatial control of neurons and myoblasts for enhancing functionality of neurons	[125]
DLP	Poly (glycerol sebacate) acrylate	C2C12	Rat VML TA	Tuning of mechanical and geometrical cues through 3D printing process	[126]
Embedded printing	GelMA	hiPSC-MPCs/HUVEC	Mouse VML quadriceps	Forming endothelialized perfusable channels with increasing iPSC-MPC’s viability and functionality	[123]

### Bioprinting

**Fig. 3:**
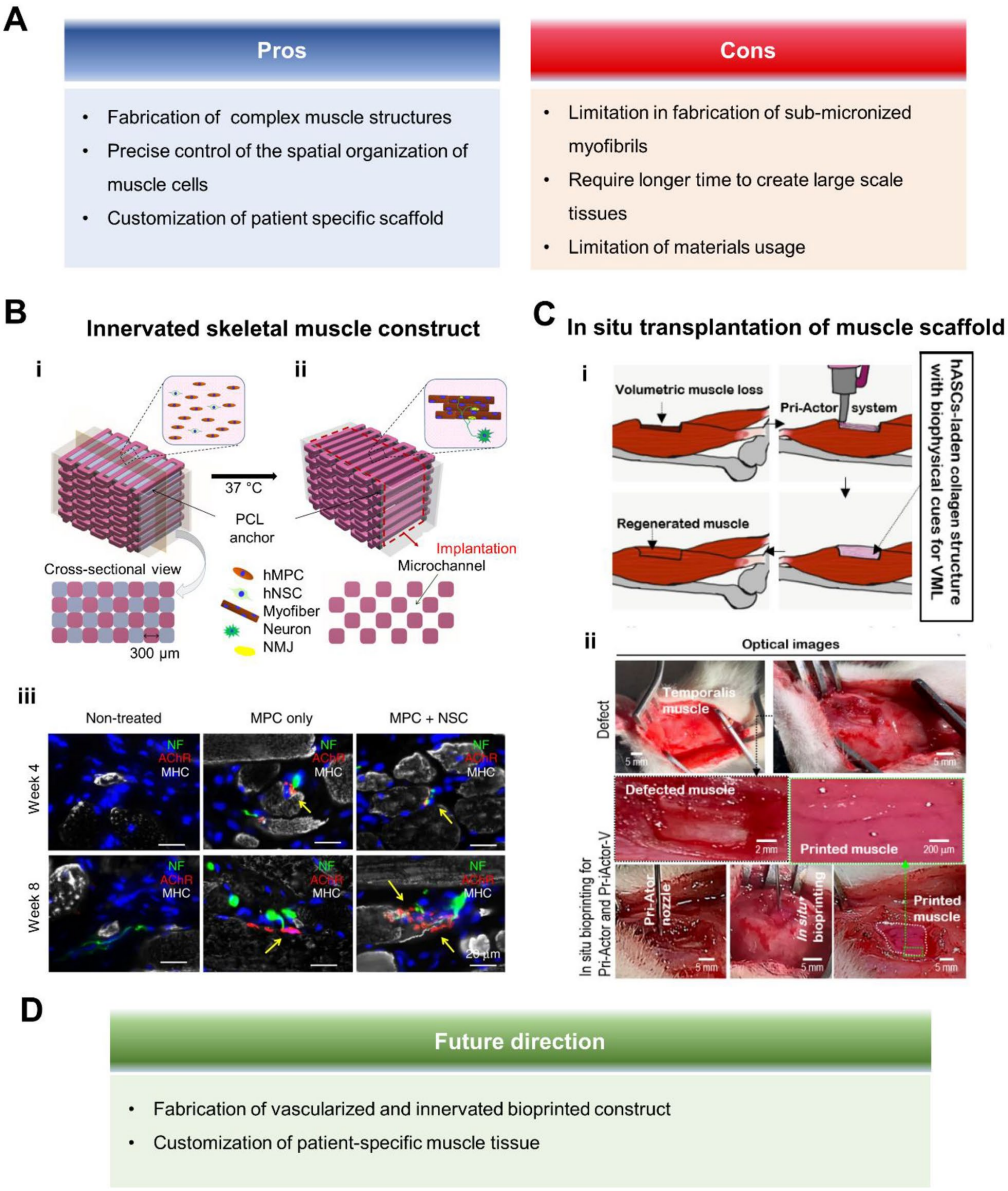
Recent bioprinting strategy for the generation of functional skeletal muscle construct. **A**. Pros and cons of bioprinting technique for fabricating skeletal muscle tissue. **B**. Neural cell integration with bioprinted skeletal muscle constructs. (i) The bioprinted construct's design, where the multi-dispensing units are applied with the acellular sacrificial bioink, the cell-laden bioink carrying hMPCs and/or hNSCs, and the supporting PCL structure. (ii) Microchannels to preserve the viability of printed cells in the structures made after the sacrificial designs were removed. (iii) MPC + NPC group showed developed neuromuscular junctions and neuronal contact on the freshly created myofibers in the transplanted constructs. (Neurofilaments; NF (green)/Acetylcholine receptor; AChR (red)/Myosin heavy chain; MHC (white)) [121]. **C**. In-situ bioprinting of hASC-laden bioink for muscle regeneration. (i) Schematic of directly printed hASC-laden collagen structure on damaged human skeletal muscle using the bioprinter-actuator combined system. (ii) Images of *in-situ* bioprinting using a bioprinter-actuator combined system in rat temporalis muscle VML model [145]. **D**. Future direction for generating functional skeletal muscle tissue

The use of 3D printing technology in tissue engineering has shown promising results for skeletal muscle regeneration. This approach involves using additive manufacturing techniques to create complex muscle structures that mimic the native tissue's design and function [115] (Fig. 3A). Bioprinting has been used to print muscle constructs using myoblast cell lines like C2C12, but more recently, stem cells have been used for muscle regeneration and repair [116, 117]. Bioprinting enables precise control of the spatial organization of cells, allowing for improved tissue engineering outcomes. In one study, inkjet bioprinting was used to engineer a spatially defined microenvironment for primary muscle-derived stem cells, promoting their differentiation into osteogenic or myogenic cells [118].

The bioprinting strategy for muscle regeneration involves developing new printing methods or printing cells that interact with muscle, such as neural cells or endothelial cells, in a spatially heterogeneous manner. For example, Kang et al*.* [119] developed the integrated tissue-organ printer (ITOP), which allows for the production of human-scale tissue. They used this technology with fibrinogen (fib), gelatin, hyaluronic acid (HA) and glycerol mixed bioink to construct a biomimetic implantable human skeletal muscle construct made up of tightly packed, viable, and aligned human primary muscle progenitor cells (hMPCs) [119, 120]. In another study, ITOP was used to create neuro-muscular junctions between hMPCs and human neural stem cells (hNSCs) with the same bioink as above, promoting rapid innervation in a rodent model of muscle defect injury [121] **(**Fig. 3B). When applied to a rat TA muscle defect model, about 1 $\times$ 10^5^ hNSCs were bioprinted with hMPCs in the middle third of the TA, demonstrating successful innervation over a span of 8 weeks. The addition of neurotrophic factors in a PLGA microsphere further accelerated nerve regeneration and innervation in hMPCs bioprinted muscle constructs [122]. Multi-material bioprinting was used to replicate muscle structural integrity by depositing perfusable vasculatures and aligned hiPSC-MPC channels within an endomysium-like supporting gel [123]. Human umbilical vein endothelial cell (HUVEC) at a density of 4 $\times$ 10^7^ cells were bioprinted within the gelatin to create a perfusable endothelialized microchannel. After implantation subcutaneously for 4 weeks, the structure sustained the viability and function of the muscle cells in vivo This resulted in a high degree of alignment and effective formation of myotubes in 1 cm^3^ hexahedral GelMA. Another technique called assembled cell-decorated collagen (AC-DC) bioprinting was invented to generate collagen microfibers with 100 μm diameter coated with MPCs or hMSCs resulting in a ring shaped structure around 10 mm in diameter, which had almost 1000 times higher tensile strength than normal collagen gels [124]. Digital light processing (DLP) and stereolithography (SLA) bioprinting were also used to precisely control (~ 5 μm) the location of heterogenous cells [125, 126]. Finally, acellular 3D bioprinted urinary bladder matrix dECM patches (≈ 12 × 8 × 2 cm) were used for implantation of a bulk patient-specific scaffold based on CT imaging, allowing for precise adaptation to complex wounds [127].

The electroconductive nature of nanomaterials, such as graphene and its family, facilitates the upregulation of myogenic gene expression in myoblasts. For example, in a study by Jo et al. [128], GO-incorporated electroconductive polyacrylamide hydrogels could enhance myogenic gene expression in myoblasts through cellular interactions with the electrical and mechanical signals provided by the nanomaterials. This unique GO substance could be easily incorporated into bioinks due to its hydrophilic nature, excellent water solubility, and easy chemical modification. For examples, the incorporation of GO as a component for a myogenesis-inducing material, into phenol-rich gelatin hydrogels used for 3D printing has been shown to improve thermal stability, increase molecular interactions, and potentially influence the patterning process during bioprinting, making the printing process more efficient [129]. Later, 3D printed GO/phenol-rich gelatin hydrogel patterns provided suitable microenvironments for improving myogenic differentiation of C2C12 cells, showing potential for muscle tissue engineering and regenerative medicine. C2C12 cells were also mixed with gold nanowires in collagen bioink, printed and then exposed to electric fields to align the nanowires [130]. This resulted in a high degree of alignment and effective formation of myotubes.

### In-situ bioprinting

*In-situ* bioprinting technology has been extensively explored to create complex and heterogeneous architectures of engineered constructs directly following their sophisticated deposition in custom designed patterns to fill cutaneous injured area [131–135]. Theses *in-situ* printing technologies have the potential to provide improved tissue regeneration ability for individual patients compared to *ex-situ* printed implants due to benefits proffered by the natural cellular microenvironment of the body [136–139] and from the ability to fabricate customized acellular or cellular scaffolds that fit different injured area and shapes on individual patients [131–134]. In recent advancements of *in-situ* bioprinting, there is a clear inclination towards incorporating novel tools to enhance the precision and adaptability of the printing process. For examples, robotic and handheld bioprinting devices are being increasingly utilized for targeted deposition of bioinks, especially, in areas requiring wound, skin, bone, muscle, and cartilage regeneration [137, 138]. The integration of camera systems and scanners provides real-time feedback, ensuring accurate placement and alignment of printed tissues [140]. The emergence of multi-degree-of-freedom bioprinting robots, equipped with advanced sensing and imaging capabilities, highlighted the intersection of robotics, imaging, and bioprinting in addressing intricate challenges, such as hair-follicle-inclusive skin repair [141]. This integration signifies the growing role of *in-situ* bioprinting within the broader context of regenerative medicine and tissue engineering.

*In-situ* bioprinting has been highlighted as a core methodology for muscle regeneration due to the feasible extensive processing times and post-bioprinting manipulations. Quint et al. [142] developed a VEGF-releasing nanomaterials-based bioink printed using a portable *in-situ* printer. The bioink incorporated laponite, an artificially manufactured nano-structure, to control the release of VEGF, enhance its adhesion to the muscle surface, increase the rheological behavior of bioinks, and increase mechanical properties due to entanglement with GelMA. This approach synergistically modulated the wound environment, leading to improved functional muscle recovery after VML in a murine model. In a related study by the same group, C2C12 myoblasts encapsulated in a GelMA bioink were directly printed onto a VML murine model (79 ± 7.5 mg resection) injury site using a partially-automated handheld bioprinter [143]. The results showed the formation of multinucleated myotubes 24 days post-printing, indicating the promising potential of *in-situ* bioprinting in muscle regeneration [144]. *In-situ* bioprinting of human ADSCs (hADSCs) with collagen in the VML area was achieved using a bioprinter combined with a bioreactor called Pri-Actor [145] (Fig. 3C). Myogenic differentiation of hADSCs was induced by the mechanical stimulation in the bioreactor, leading to the formation dense myofibers in VML model of rat in the temporalis muscle. *In-situ* bioprinting represents a significant step forward in regenerative medicine, showing potential in muscle regeneration.

### Other engineered 3D muscle tissues

Various engineered 3D muscle tissues, through molding and microfluidics have been used, in addition to bioprinting, to produce muscle constructs. Molding is a popular method for creating 3D muscle tissue using stem cells and hydrogels. Collagen and fibrinogen are commonly used polymers, as they are easily crosslinked. The type of biomaterial used does not significantly affect the differentiation of stem cells [76]. For instance, a mixture of collagen and hMPCs was used to create a 3D structure for laryngeal reconstruction [146]. Molding is not only used for implantation but also for in vitro models, and can be used to study the effects of culture media on hPSC myogenesis and contractile force, or as an intramuscular injection model for drug testing [147, 148]. In addition, fiber-shaped molds can be used to create single fibers with a length of 10 mm and diameter of 120 μm. When ESC-derived myoblasts were placed in Matrigel inside a mold, muscle maturation could be observed within 7 days [149].

Microfluidic devices have been used as models to study the interactions between different tissues, including muscle tissue. Osaki et al*.* [150] used a microfluidic chip to culture muscle bundles and iPSC-derived motor neuron spheroids in different compartments to create an amyotrophic lateral sclerosis (ALS) model. In the chip-based ALS motor unit, fewer muscle contractions and muscle apoptosis were observed. Microfluidic chips offer a promising platform for assessing the safety of nanomaterials in muscle regeneration. Their inherent ability to produce laminar flow ensures a uniform exposure of nanomaterials to cells [151]. Additionally, by manipulating the flow rate, there is precise control over the cellular uptake of these nanomaterials [152]. Given their successful applications in skin models, it is anticipated that microfluidic chips will find extensive utility in muscle models in forthcoming research [153].

## Conclusion and future perspectives

In conclusion, the development of skeletal muscle tissue engineering has led to promising advances in the field of regenerative medicine. As the understanding of the complex interplay among cells, materials, and mechanical forces continues to grow, new research areas will emerge, leading to the full realization of the potential of personalized, customized, and biomanufacturable platforms for muscle regeneration. Nanomaterials, indeed, offer great potential for muscle regeneration. However, the exact mechanism behind their effectiveness still requires deeper exploration for clinical translation. It is speculated that scaffolds built using nanomaterials like GO, CNT, and AuNP not only enhance mechanical and electrical properties but also influence the differentiation and proliferation of myogenic stem cells. Furthermore, safety issues related to the application of non-biodegradable nanomaterials, such as bio-accumulation or long-term exposure effects, need to be considered.

One of the most promising fabrication techniques for producing muscle scaffolds is 3D bioprinting combined with nanomaterials and nanotechnologies. This technique enables the precise positioning of acellular or cellular structures, resulting in a biomimetic scaffold that mimics the natural tissue. Additionally, computed tomography (CT)-guided bioprinting can be used to replicate the tissue microarchitecture obtained from patient CT images following an injury, leading to the creation of custom scaffolds tailored to individual patients. Once proven effective in a mouse model, custom scaffolds will undergo Phase I testing before being considered for use in human patients (Fig. 3D).

The biomanufacturing industry is another area that holds great promise in the production of functional tissues for direct implantation in patients. By using 3D bioprinting to digitally design the scaffold and establishing automated or semi-automated biomanufacturing processes, it can improve the quality of an acellular or cellular scaffold and ultimately lead to enhanced efficacy of implants and related clinical therapies. The ability to fabricate and culture tissues in a high throughput manner and in real-time can greatly improve the speed and efficiency of the biomanufacturing process, making treatments more accessible to patients in need.

Looking towards the future, there are several avenues of research that are most relevant and productive. These include exploring the use of new materials and nanotechnology for tissue engineering, developing new methods for in vitro muscle maturation and neural integration, and investigating the use of iPSCs and gene editing techniques for personalized therapies. Furthermore, advancements in artificial intelligence and machine learning can help to optimize scaffold design and improve the efficacy of clinical treatments.

In summary, the novel approaches discussed in this review have the potential to significantly enhance the patient’s quality of life. By continuing to invest in research and development in the field of skeletal muscle tissue engineering, we can accelerate the translation of these promising therapies into clinical practice, ultimately improving the lives of millions of people worldwide.

## Data Availability

Not applicable.

## Abbreviations

3D: Three-dimentional
VML: Volumetric muscle loss
ECM: Extracellular matrix
ADSCs: Adipose-derived stem cells
MSCs: Mesenchymal stem cells
hPSC: Human pluripotent stem cells
hESCs: Human embryonic stem cells
hiPSCs: Human induced pluripotent stem cells
SMCs: Skeletal muscle cells
hMPCs: Human primary muscle progenitor cells
IGF-1: Insulin-like growth factor-1
IGF-2: Insulin-like growth factor-2
TGF-β: Transforming growth factor-beta
VEGF: Vascular endothelial growth factor
HGF: Hepatocyte growth factor
FGF-2: Fibroblast growth factor-2
dECM: Decellularized ECM
PCE: Poly(citric acid-octanediol-polyethylene glycol)
MAPK: Mitogen-activated protein kinase
CNT: Carbon nanotube
ITOP: Integrated tissue-organ printer
GO: Graphene oxide
NPs: Nanoparticles
hNSCs: Human neural stem cells
HUVEC: Human umbilical vein endothelial cell
NF: Neurotrophic factor
PLGA: Poly(lactic-co-glycolic acid)
AuNPs: Gold nanoparticles
PCL: Polycaprolactone
PEG: Poly(ethylene glycol)
IL: Interleukin
TA: Tibialis anterior
Fib: Fibrinogen
BMP-2: Bone morphogenetic protein-2
SLA: Stereolithography
DLP: Digital light processing
CT: Computed tomography
AC-DC: Assembled cell-decorated collagen
LD: Latissimus dorsi
